# Antiproliferative effect of ZSTK474 alone or in combination with chemotherapeutic drugs on HL60 and HL60/ADR cells

**DOI:** 10.18632/oncotarget.16589

**Published:** 2017-03-28

**Authors:** Qianxiang Zhou, Yali Chen, Lei Zhang, Yuxu Zhong, Zhe Zhang, Ran Wang, Meihua Jin, Min Gong, Yuling Qiu, Dexin Kong

**Affiliations:** ^1^ Tianjin Key Laboratory on Technologies Enabling Development of Clinical Therapeutics and Diagnostics, School of Pharmacy, Tianjin Medical University, Tianjin, 300070, China; ^2^ Research Center of Basic Medical Sciences, Tianjin Medical University, Tianjin, 300070, China; ^3^ State Key Laboratory of Toxicology and Medical Countermeasures, Beijing Institute of Pharmacology and Toxicology, Beijing, 100850, China

**Keywords:** ZSTK474, HL60/ADR, PI3K, resistance, drug combination

## Abstract

While chemotherapy remains to be one of the main approaches in the clinical treatment of acute myeloid leukemia (AML), multidrug resistance (MDR) has become a serious problem which limits the therapeutic efficacy. The important roles of the PI3K/Akt pathway in modulating cell proliferation and MDR suggest that PI3K inhibitor might be effective for treatment of AML. In the present study, the antiproliferative effects of PI3K inhibitor ZSTK474 on AML cell HL60 and the adriamycin (ADR)-resistant HL60/ADR cells were investigated. Our data indicated that ZSTK474 exhibited potent antiproliferative activity, induced G1 cell cycle arrest, but no obvious apoptosis in both cell lines. Moreover, ZSTK474 affected the protein levels of cell-cycle-related molecules including increased p27, decreased cyclin D1 and phosphorylated Rb in dose-dependent manner. The proteins downstream of PI3K including phosphorylated PDK1, Akt and GSK-3β were reduced in a dose-dependent manner after ZSTK474 treatment. ZSTK474 reversed ADR resistance, increased the intracellular accumulation of ADR, and reduced the expression and function of multidrug resistance (MDR) proteins including both P-gp and MRP1 in HL60/ADR cells. The combination of ZSTK474 and chemotherapeutic drugs cytarabine or vincristine led to a synergistic effect in HL60 and HL60/ADR cells. In conclusion, ZSTK474 showed potent antiproliferative effect on HL60 and HL60/ADR cells; combination with cytarabine or vincristine resulted in synergistic effect. Our results suggest ZSTK474 has the potential to be applied in the treatment of AML patients, while further evidences particularly those about *in vivo* efficacy are needed.

## INTRODUCTION

Acute myeloid leukaemia (AML) is a complex disease characterized by abnormal differentiation and unlimited cellular proliferation of haematopoietic stem cells. Current clinical treatments such as chemotherapeutic drugs, radiation, and stem cell transplantation are associated with incomplete remission and side effects. Multidrug resistance (MDR) is a major obstacle to successfully treating AML patients with chemotherapy [1–3]. MDR is characterized by decreased intracellular drug accumulation after a period of drug administration, which is mediated by increase of drug efflux by ATP-binding cassette (ABC) transporters such as P-glycoprotein (P-gp) and MRP1 [4, 5]. Therefore, novel anticancer drugs that are effective on multidrug resistant AML are expected.

Phosphatidylinositol 3-kinases (PI3Ks) are lipid kinases that mainly phosphorylate phosphatidylinositol 4,5-bisphosphate (PIP_2_) and generate phosphatidylinositol 3,4,5-trisphosphate (PIP_3_), which activates the downstream Akt and thus regulates signaling pathways involved in cell proliferation and the cell cycle [6]. Previous studies have indicated that Akt activation can induce MDR in tumors such as ovarian cancer, breast cancer and hepatocellular carcinomas, suggesting that PI3K/Akt pathway might be involved in MDR developed during cancer chemotherapy [7, 8]. ZSTK474, a specific PI3K inhibitor that we previously identified by using the JFCR39 drug discovery system [9, 10], exhibited potent antitumor activity against various tumors [11, 12]. However, the effect of ZSTK474 on AML and MDR has not yet been reported.

Therefore, we recently investigated the vitro antitumor activities of ZSTK474 toward AML as well as the ability to reverse MDR. Moreover, we evaluated the synergistic effects of ZSTK474 in combination with chemotherapeutic drugs.

## RESULTS

### ZSTK474 inhibited proliferation of HL60 and HL60/ADR cells

**Figure 1:**
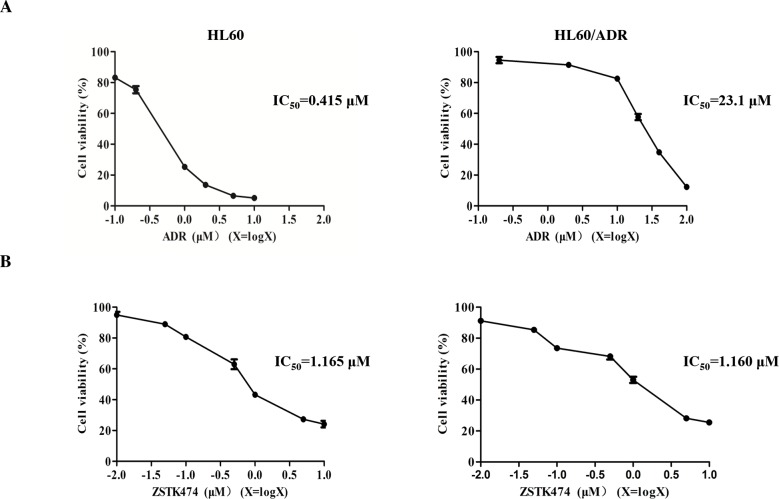
Antiproliferative activity of ZSTK474 in HL60 and HL60/ADR cells **(A)** HL60/ADR cells showed resistance to ADR. Cell viability was determined via MTT assay after treatment with various concentrations of ADR for 48 h. **(B)** ZSTK474 inhibited the proliferation of both HL60 and HL60/ADR cells in a dose-dependent manner. The cells were treated with various concentrations of ZSTK474 for 48 h, and cell viability was determined by MTT assay. Data are presented as mean ± SD and are representative of three independent experiments.

HL60 is a chemosensitive cell line, whereas HL60/ADR cells are resistant to ADR as well as other chemotherapeutic drugs. As shown in Figure 1A, IC_50_ of ADR for HL60/ADR is 23.1 μM while that for HL60 is 0.415 μM. The relative resistance (RR) was therefore calculated to be 55.6, confirming that the former cell line indeed showed resistance to ADR [13]. We then investigated the antiproliferative effects of ZSTK474 on both cell lines by using MTT assay. ZSTK474 inhibited proliferation of both HL60 and HL60/ADR cells in a dose-dependent manner, with IC_50_ as 1.165 μM and 1.160 μM (Figure 1B), respectively. Cell count assay indicated similar result (Supplementary Figure 1). The similar antileukaemia potency of ZSTK474 on HL60 and HL60/ADR cells suggested that ZSTK474 might not be a substrate of MRP1/ P-gp in HL60/ADR cells.

**Figure 2:**
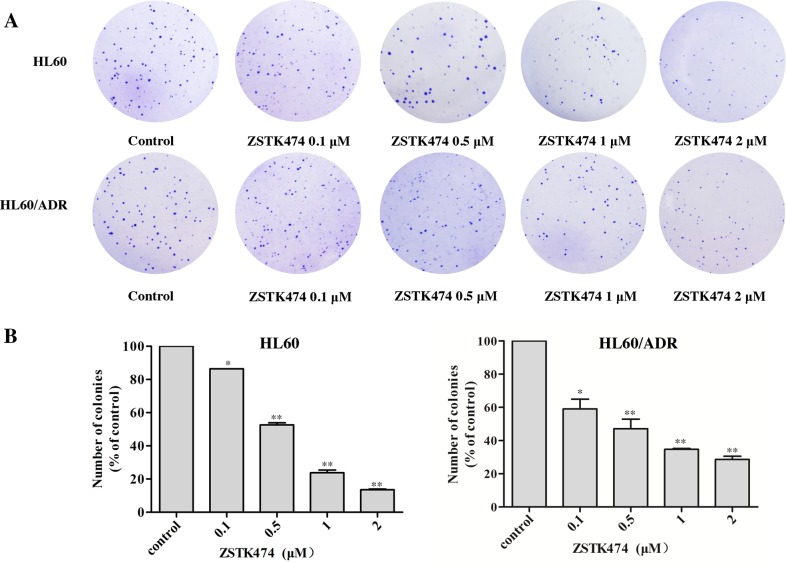
ZSTK474 inhibited colony formation by HL60 and HL60/ADR cells **(A)** HL60 and HL60/ADR cells were pre-treated with various concentrations of ZSTK474 for 48 h and cultured in soft agar for 14 days. The colonies formed were observed and photographed with microscope. **(B)** The relative colony numbers were quantified and normalized to those of control cells (without treatment). The results represent mean ± SD of three independent experiments. *: P<0.05, **: P<0.01, ***: P<0.001, compared with control.

Next, the colony formation assays were conducted to further examine the effects of ZSTK474 on HL60 and HL60/ADR proliferation. The cells were pre-treated with 0, 0.1, 0.5, 1 or 2 μM of ZSTK474 for 48 h and grown in soft agar for 14 days. As indicated in Figure 2A and 2B, the number of the colonies formed by ZSTK474-treated cells was reduced markedly compared with control (without treatment), in either HL60 or the resistant HL60/ADR cells.

### ZSTK474 induced G1 cell cycle arrest, but did not induce apoptosis in HL60 and HL60/ADR cells

**Figure 3:**
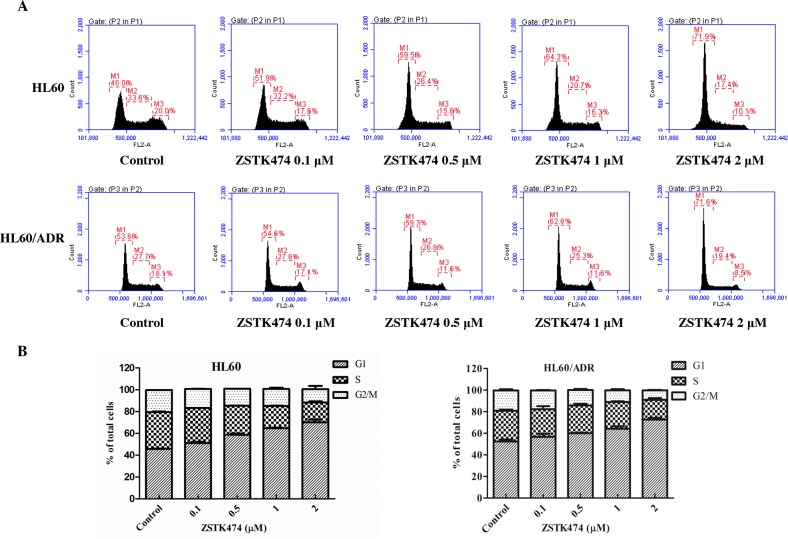
ZSTK474 induced cell cycle arrest at G1 phase in HL60 and HL60/ADR cells **(A)** Cell cycle distribution analysis. HL60 and HL60/ADR cells were incubated with indicated concentrations of ZSTK474 for 48 h. The cells were harvested to be available for flow cytometry. **(B)** The percentages of the cell population in G1, S, and G2/M phases were determined. Data are presented as mean ± SD, representative of three independent experiments.

**Figure 4:**
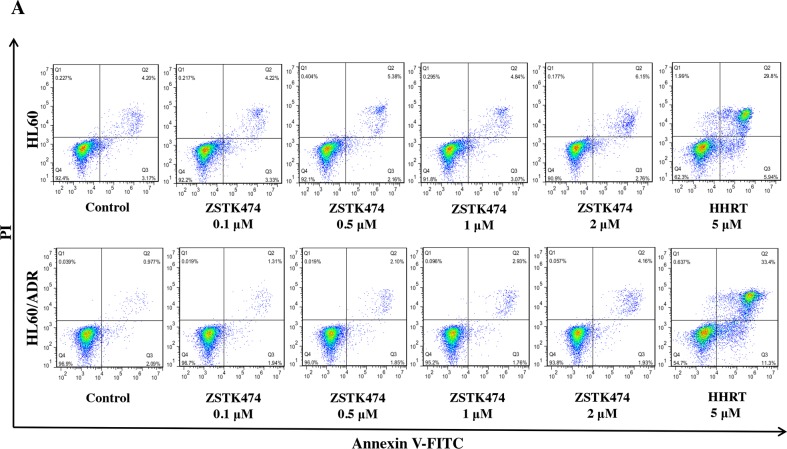

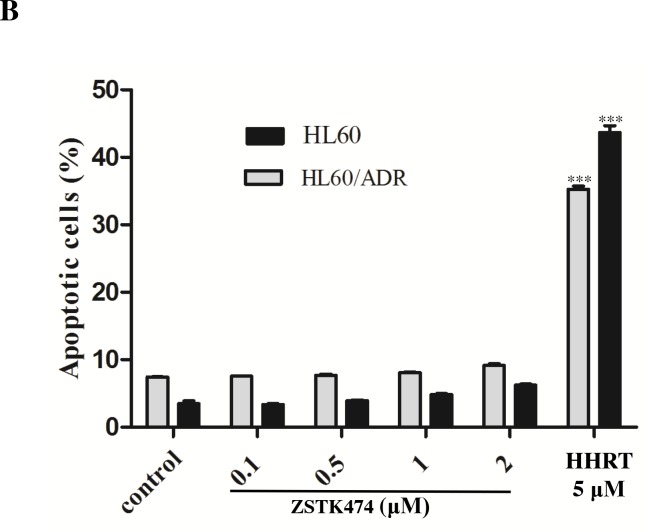
ZSTK474 did not induce obvious apoptosis in HL60 and HL60/ADR cells **(A)** HL60 and HL60/ADR cells were incubated with the indicated concentrations of ZSTK474 for 48 h. The cells were harvested, double stained with annexin V and PI, and analyzed with flow cytometry. Homoharringtonine (5 μM) was used as a positive control. **(B)** The percentage of the apoptotic cell population was determined. Data are presented as mean ± SD, representative of three independent experiments.***: P<0.001, compared with control.

Because cell cycle progression is necessary for cell proliferation, we investigated the effect of ZSTK474 on cell cycle of HL60 and HL60/ADR cells. The cells were treated with 0, 0.1, 0.5, 1 or 2 μM of ZSTK474 for 48 h, and analyzed by flow cytometry with propidium iodide (PI) staining. The results showed that ZSTK474 induced G1 arrest in both HL60 and HL60/ADR cells in a dose-dependent manner (Figure 3A and 3B). On the other hand, as shown in Figure 4A and 4B, the annexin V/PI staining assay did not detect apparent apoptosis in ZSTK474-treated HL60 or HL60/ADR cells, suggesting that the antiproliferative activity of ZSTK474 might be unrelated to apoptosis induction. Homoharringtonine (HHRT) was used as a positive control.

### ZSTK474 affected the cell cycle-related molecules in HL60 and HL60/ADR cells

**Figure 5:**
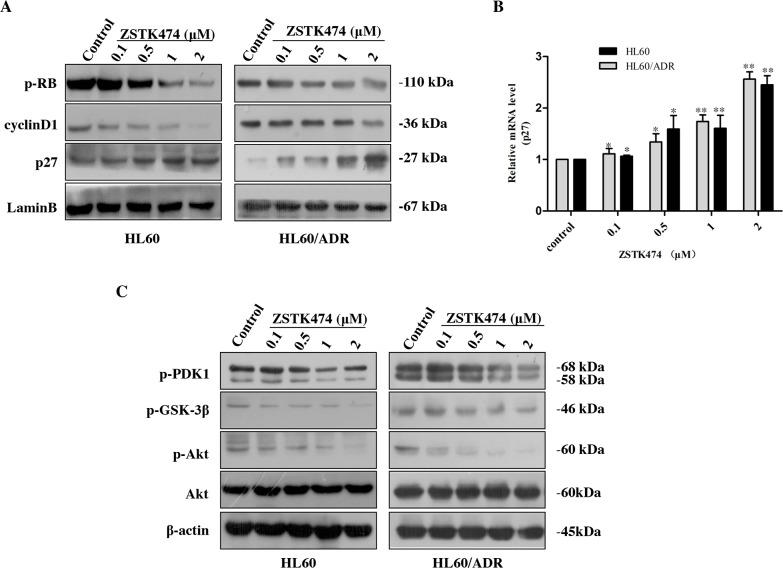
ZSTK474 affected the cell cycle-related molecules **(A)** Western blot analysis of cell cycle-related proteins after ZSTK474 treatment. HL60 and HL60/ADR cells were treated with indicated concentrations of ZSTK474 for 48 h. The levels of cyclin D1, p27, and p-pRb in the nuclei were determined. **(B)** Quantitative RT-PCR analysis of p27 mRNA expression. The relative gene expression levels were quantified by using the comparative Ct (ΔΔCt) method. The results represent mean ± SD of three independent experiments. *: P<0.05, **: P<0.01, ***: P<0.001, compared with control. **(C)** ZSTK474 inhibited PI3K/Akt pathway in HL60 and HL60/ADR cells. HL60 and HL60/ADR cells were cultured in the presence of various concentrations of ZSTK474 for 48 h. The cells were collected, and the cell lysates were prepared to be available for Western blot analysis of p-PDK1, p-Akt, Akt, p-GSK-3β, and β-actin levels.

Cell cycle progression is promoted by CDK (cyclin-dependent kinase)-cyclin complexes and negatively regulated by CDK inhibitors such as p27. To investigate the mechanism of G1 arrest by ZSTK474, we analyzed the amounts of the proteins known to be involved in G1/S progression, including cyclin D1, p27 and downstream p-Rb. As shown in Figure 5A, the expression of p27 increased, whereas the levels of cyclin D1 and phosphorylated Rb decreased dose-dependently. The effect of ZSTK474 on p27 mRNA expression was also examined via quantitative real-time polymerase chain reaction (qRT-PCR). Figure 5B shows that the RNA expression levels of p27 in HL60 and HL60/ADR cells clearly enhanced after ZSTK474 treatment. The results suggest that an increase of p27 expression, as well as reduction of cyclin D1 expression and Rb phosphorylation might be involved in ZSTK474-induced G1 arrest. Since ZSTK474 is a specific PI3K inhibitor, to correlate the G1 arrest effect to its inhibition against PI3K/Akt pathway, we examined the effects of ZSTK474 on the proteins downstream of PI3K. Figure 5C shows that the levels of phosphorylated PDK1, Akt and GSK-3β were reduced in a dose-dependent manner after ZSTK474 treatment.

### ZSTK474 reversed ADR resistance in HL60/ADR cells

Since the antiproliferative potency of ZSTK474 on HL60/ADR cells is similar to that on the parent HL60 cells, we then assessed whether ZSTK474 could reverse the MDR resistance in HL60/ADR cells. We first examined the resistance to ADR of the cells in the presence of ZSTK474. As shown in Table 1, ADR alone displayed weak inhibition against proliferation of HL60/ADR cells with IC_50_ as 23.1 μM. However, co-treatment with ZSTK474 reduced the resistance to ADR in a dose-dependent manner, with the reversal fold (RF) values to be 1.34, 5.31, 29.9 and 30.4, for 0.1, 0.5, 1 and 2 μM of ZSTK474, respectively.

**Table 1:** Effect of ZSTK474 on reversing ADR resistance in HL60/ADR cells

Compounds		IC50(μM) (fold-reversal)
ADR (μM)	ZSTK474 (μM)	
0.2-100.0	0	23.1 (1.00)
	0.1	17.2 (1.34)
	0.5	4.35 (5.31)
	1	0.772 (29.92)
	2	0.761 (30.35)

### ZSTK474 increased the intracellular accumulation of ADR

**Figure 6:**
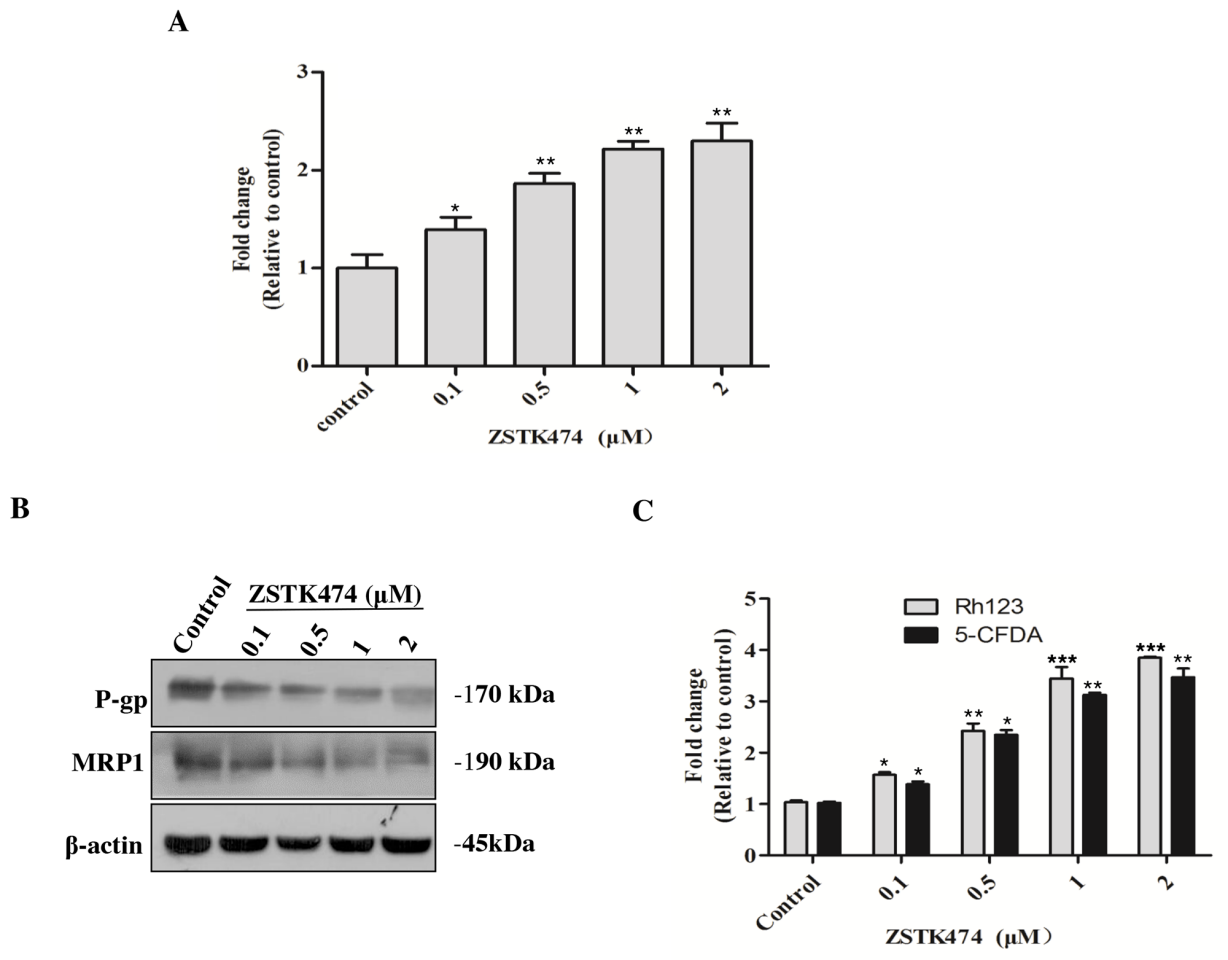
ZSTK474 increased the intracellular accumulation of ADR via inhibiting MDR proteins in HL60/ADR cells **(A)** ZSTK474 increased intracellular accumulation of ADR. HL60/ADR cells were incubated with 10 μM of ADR in the presence of indicated concentrations of ZSTK474 for 3 h. The data were quantified by comparing the fluorescence values to those of the control (without ZSTK474 treatment). **(B)** ZSTK474 inhibited expression of P-gp and MRP1. HL60/ADR cells were cultured in the presence of indicated concentrations of ZSTK474 for 48 h. The cells were collected for Western blot analysis of P-gp and MRP1 levels. **(C)** ZSTK474 increased intracellular accumulation of P-gp substrate Rh123 and MRP1 substrate 5-CFDA. HL60/ADR cells were pre-treated with Rh123 and 5-CFDA for 0.5 h, washed and further incubated with indicated concentrations of ZSTK474 for 3 h. Fluorescence intensity was measured with flow cytometry The data were quantified by comparing the fluorescence values to those of control. The results represent mean ± SD of three independent experiments. *: P<0.05, **: P<0.01, ***: P<0.001, compared with control.

To investigate the mechanism of reversal effect of ZSTK474 on resistance to ADR, we determined the intracellular accumulation of ADR in HL60/ADR cells with or without ZSTK474 treatment. As shown in Figure 6A, ZSTK474 increased intracellular accumulation of ADR in a dose-dependent manner, indicating that ZSTK474 reversed drug resistance by reducing ADR efflux from HL60/ADR cells.

### ZSTK474 inhibited the expression and function of MDR proteins

Then we analyzed the effect of ZSTK474 on MDR proteins in HL60/ADR cells. As shown in Figure 6B, the expression of both P-gp and MRP1 protein was decreased in a dose-dependent manner after ZSTK474 treatment. To confirm the effect on the functions of MDR proteins, the intracellular accumulation of P-gp substrate Rh123 and MRP1 substrate 5-CFDA in HL60/ADR cells was determined. As shown in Figure 6C, the accumulation of both Rh123 and 5-CFDA was increased in a dose-dependent manner, as compared with that in the cells without ZSTK474 treatment, further supporting that ZSTK474 might reverse drug resistance by negatively affecting the expression and function of MDR proteins.

### Antiproliferative effects of ZSTK474 in combination with chemotherapeutic drugs on HL60 and HL60/ADR cells

**Figure 7:**
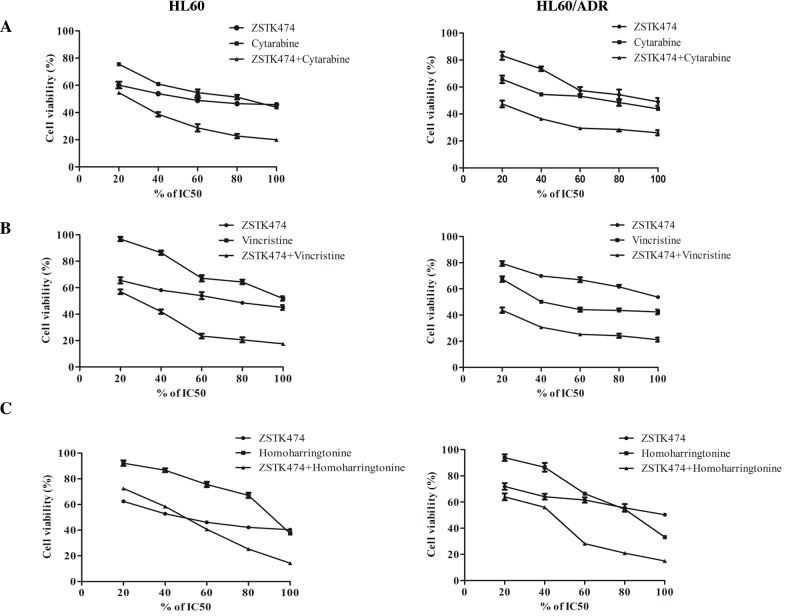

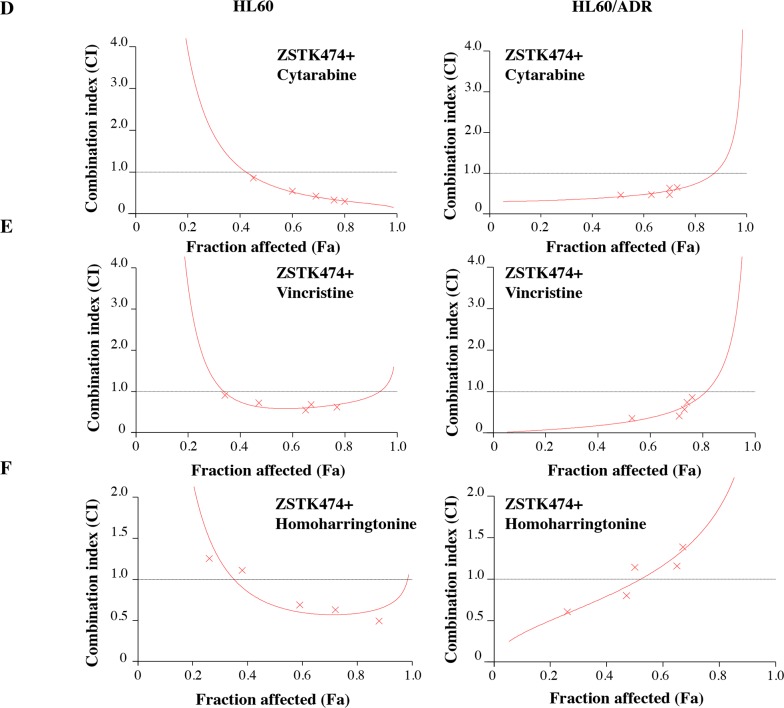
Antiproliferative effect of ZSTK474 in combination with chemotherapeutic drugs on HL60 and HL60/ADR cells **(A-C)** HL60 (left) and HL60/ADR (right) cells were incubated with various concentrations of ZSTK474 and chemotherapeutic drugs (20%, 40%, 60%, 80%, 100% IC_50_ of each drug), either alone or in combination. Cell viability after each treatment was determined by MTT assay. Data are presented as mean ± SD, representative of three independent experiments. **(D-F)** Combinational effect was analyzed using CalcuSyn software and the resulting CI-Fa plots are shown for HL60 and HL60/ADR cells. The horizontal line of CI = 1, representing additivity, is indicated. Values of drug combinations below the horizontal line indicate synergism. CI: combination index, Fa: fraction affected.

Finally, we investigated the antiproliferative effects of ZSTK474 on HL60 and HL60/ADR cells in combination with various chemotherapeutic drugs. Drug combinations were carried out by adopting a fixed ratio of ZSTK474 to chemotherapeutic drugs (1×IC_50 ZSTK474_ : 1×IC_50 chemotherapeutic drug_) and using a series of drug concentrations at this fixed ratio (20%, 40%, 60%, 80% and 100% of the IC_50_value of each drug). Figure 7A–7C shows that co-treatment with ZSTK474 led to greater inhibition of cell proliferation than each chemotherapeutic drug alone. Combination of ZSTK474 with cytarabine and vincristine exhibited a synergistic effect at all of the tested concentrations with CI<1 (Figure 7D–7E). However, combination with homoharringtonine does not always result in synergistic effect in either HL60 cells or HL60/ADR cells (Figure 7F).

## DISCUSSION

AML is a heterogeneous disease of the blood originating in the bone marrow. Aberrant activation of the PI3K/Akt/mTOR pathway is a common feature of AML [14]. This pathway has key functions in regulating cell growth, survival and cell cycle. Furthermore, different groups have demonstrated that PI3K/Akt/mTOR activation is an indicator of chemoresistance in AML [15–17]. ZSTK474 is a specific class I PI3K inhibitor we previously identified by using the JFCR39 drug discovery system [10]. In the present study, we investigated for the first time the efficacy of ZSTK474 on AML. ZSTK474 inhibited the proliferation of HL60 and HL60/ADR cells with IC_50_ value of 1.165 μM and 1.160 μM, respectively. Cell count assay and colony formation assay demonstrated that ZSTK474 potently inhibited the proliferation and tumorigenicity of HL60 and HL60/ADR cells.

Flowcytometric analysis with PI staining indicated that ZSTK474 induced G1 arrest in both HL60 and HL60/ADR cells. Cell cycle progression is known to be promoted by cyclin-CDK complexes and inhibited by CDK inhibitor proteins such as p27. Treatment with ZSTK474 increased p27 level but decreased cyclin D1 and p-Rb levels. GSK-3β (a downstream effector of Akt) has been reported to increase cyclin D1 expression [18], and Akt is known to upregulate p27 [19]. Phosphorylation of Akt and GSK-3β was inhibited by ZSTK474, thus supporting the notion that blocking PI3K/Akt signaling might be involved in G1 arrest. On the other hand, our results revealed that treatment with ZSTK474 for 48 h only induced slight apoptosis in HL60 and HL60/ADR cells, suggesting that ZSTK474 exhibits antitumor effects mainly via G1 arrest but not apoptosis which is consistent with the results reported previously [20, 21].

Emergence of MDR is the greatest obstacle to successful implementation of chemotherapy in cancer patients. In recent years, the relationship between ABC transporter expression and PI3K/Akt pathway has received much attention [8, 22–24]. Particularly, it has been reported that activation of PI3K/Akt pathway could promote the transcription and expression of MRP1 and MDR1 (the gene that encodes P-gp) genes, via affecting downstream transcription factors Nrf2 (NF-E2-related factor 2) and NF-κB, respectively [25, 26]. Our results revealed that 2 μM of ZSTK474 strongly reversed ADR resistance by over 30-fold. Meanwhile, ZSTK474 decreased P-gp and MRP1 protein expression in a dose-dependent manner. And the function of P-gp and MRP1 as the MDR-relevant efflux pumps was also inhibited by ZSTK474. At similar concentrations, ZSTK474 decreased the levels of Akt phosphorylation, suggesting that blockade of PI3K/Akt pathway might contribute to the resistance reversal activity of ZSTK474.

As common clinical antileukaemia drugs, cytarabine, vincristine and homoharringtonine can provide brief remission to patients with AML. However, these highly toxic drugs can be offered to only a subset of the patients [27, 28]. Since proper drug combination often leads to higher efficacy than that of a single drug, with a limited toxicity [29], we tested the antileukaemia activity of each of the 3 chemotherapeutic drugs in combination with ZSTK474. Our results showed that the antiproliferative effect of either cytarabine or vincristine on HL60 and HL60/ADR cell was obviously increased when combined with ZSTK474, and these combinations were synergistic. Combination of homoharringtonine with ZSTK474 led to a better effect compared with that with homoharringtonine alone, but the combination was not synergistic, of which the mechanism remains to be elucidated. To the best of our knowledge, this is the first report for combination effect of PI3K inhibitors and chemotherapeutic agents in AML cells.

In conclusion, ZSTK474 exhibited antileukaemia activity by inducing G1 arrest, and reversed the acquired drug resistance by inhibiting P-gp and MRP1 expression via the PI3K/Akt pathway. A synergistic effect was observed in the AML HL60 cell line as well as the multidrug resistant HL60/ADR cell line when combined with chemotherapeutic drugs cytarabine or vincristine. Our findings suggest the potential application of ZSTK474 in AML therapy while *in vivo* evidences are still required.

## MATERIALS AND METHODS

### Reagents

ZSTK474, adriamycin (ADR), cytarabine, vincristine and homoharringtonine were obtained from Selleck (London, ON, Canada). MTT (3-(4, 5-dimethyl-2-thiazolyl)-2, 5-diphenyl-2-H-tetrazolium bromide) reagent was purchased from Amresco (Solon, OH, USA). Antibodies against phospho-PDK1 (Ser241), Akt, phospho-Akt (Ser473), phospho-GSK-3β (Ser9), β-actin, as well as anti-mouse and anti-rabbit HRP-conjugated secondary antibodies, were purchased from Cell Signaling Technology (Danvers, MA, USA). A FITC Annexin V Apoptosis Detection Kit, antibodies against p-Rb (pS780), cyclin D1 and p27 were purchased from BD Biosciences (San Jose, CA, USA). Antibodies against P-gp, MRP1 and Lamin B were from Santa Cruz Biotechnology (Santa Cruz, CA, USA). Rhodamine123 (Rh123) and 5-carboxyfluorescein diacetate (5-CFDA) were purchased from Sigma-Aldrich (St. Louis, MO, USA).

### Cell culture

The human acute myeloid leukaemia HL60 cell line was purchased from the Cell Resource Centre, Peking Union Medical College (Beijing, China). HL60/ADR was obtained from the Institute of Haematology, Chinese Academy of Medical Sciences (Tianjin, China). Cells were cultured in RPMI 1640 medium supplemented with 20% (v/v) fetal bovine serum, 1% kanamycin (100 μg/ml) and 1% glutamine (0.44 μg/ml) in a 5% CO_2_ incubator at 37°C. ADR (final concentration as 0.5 μg/ml) was added to the medium to maintain the MDR phenotype in the HL60/ADR cells. The cells were further cultured in ADR-free medium for 2 weeks before experiments.

### Cell proliferation and colony formation assay

Assessment of cell proliferation was performed using MTT assays, as described in our previous reports [30, 31]. Briefly, 200 μl of cell suspension (2×10^4^ cells/ml) was seeded in each well of a 96-well plate and treated with various concentrations of ZSTK474 for 48 h at 37°C. After the addition of MTT, the cells were incubated for an additional 4 h. Then, the culture medium was removed, and the purple formazan crystals were dissolved DMSO. The resulting absorbance at 490 nm was measured by using a microplate reader iMark (BIO-RAD, Hercules, CA, USA).

For the colony formation assay, pre-treated cells were resuspended in 2 ml of agarose solution (0.4%) in complete medium as the upper agar layer and seeded into 60 mm dishes in which the bottom agar layer comprised of 2 ml of complete medium and agarose solution (0.8%) had already solidified. After incubation for 14 days, the colonies were fixed with 4% paraformaldehyde, stained with 0.5% crystal violet, and the number of colonies was counted. The experiments were performed in triplicate and repeated three times.

### Flow cytometric analysis of cell cycle distribution and apoptosis

Assessment of cell cycle distribution was performed by flow cytometry analysis as previously described by us [32]. Briefly, 2 ml of cell suspension (5×10^5^ cells/ml) was seeded in a 6-well plate. After treatment with 0, 0.1, 0.5, 1 and 2 μM of ZSTK474 for 48 h, cells were collected, washed with ice-cold PBS and fixed with 70% ethanol overnight at 4°C. The cell suspension was centrifuged, and the cell pellet was resuspended in 25 μg/ml of PI solution containing 0.5% Triton X-100 and 2% RNase A. The treated cells were incubated for 30 minutes in the dark at 4°C and analyzed with a BD Accuri C6 flow cytometer (BD Biosciences, San Jose, CA, USA).

Annexin V and PI staining assays were conducted to detect apoptosis induced by ZSTK474 as we described previously [12, 33]. A FITC Annexin V Apoptosis Detection Kit was used according to the manufacturer's protocol. HL60 and HL60/ADR cells were treated with different concentrations of ZSTK474 for 48 h. Then, the cells were collected, washed twice with cold PBS and resuspended in 1×binding buffer. Approximately 10^5^ cells were stained with 2.5 μl of Annexin V-FITC and 2.5 μl of PI in 100 μl of binding buffer for 15 min at room temperature in the dark, followed by analysis with a FACS Verse flow cytometer (BD Biosciences, San Jose, CA, USA).

### Determination of reversal fold values

Determination of reversal fold values was performed using MTT assay. Cell suspension was seeded in each well of a 96-well plate and treated with various concentrations of ADR in combination with ZSTK474 (0, 0.1, 0.5, 1, 2 μM) for 48 h at 37°C. The resulting absorbance at 490 nm was measured by using a microplate reader iMark as described above.

### Determination of intracellular accumulation of ADR

HL60/ADR cells were seeded at a density of 1×10^5^ cells in a 6-well plate and incubated in the presence of either ADR (10 μM) alone or in combination with ZSTK474 (0.1, 0.5, 1 and 2 μM) for 3 h at 37°C [25, 34]. Cells were collected and washed twice with cold PBS, and the mean intracellular fluorescence intensity associated with ADR was measured with a flow cytometer at an excitation wavelength of 488 nm and an emission wavelength of 575 nm.

### Determination of intracellular accumulation of Rh123 and 5-CFDA

The fluorescence intensity of intracellular Rh123 and 5-CFDA accumulation was determined by flow cytometry as reported [5, 35]. Cells were harvested, washed with PBS and incubated with the P-gp substrate Rh123 (0.05 μM) or MRP1 substrate 5-CFDA (0.05 μM) for 0.5 h at 37°C in the dark. Then, the cells were collected, washed, and further incubated at 37°C in the presence of 0, 0.1, 0.5, 1 and 2 μM of ZSTK474. After 3 h, the cells were washed again, and the fluorescence was determined by flow cytometry analysis at an excitation wavelength of 488 nm and an emission wavelength of 535 nm.

### Western blot analysis

Western blot analysis was conducted as we previously reported [36, 37]. Total protein and nuclear protein were exacted from HL60 and HL60/ADR cells by using a RIPA lysis buffer-based protocol (Roche Diagnostics, Basel, Switzerland) and a NE-PER Nuclear and Cytoplasmic Extraction Kit (Thermo Fisher Scientific, Waltham, MA, USA), respectively. Protein concentrations were quantified by using a BCA Protein Assay Kit (Pierce, Rockford, IL, USA). Equal amounts of protein were subjected to 10% SDS-polyacrylamide gel electrophoresis (PAGE) and transferred to a PVDF membrane (Millipore, Billerica, MA, USA). The membrane was blocked in 5% non-fat dry milk for 1 h, probed with the specified primary antibodies overnight at 4°C and subsequently exposed to respective anti-mouse or anti-rabbit secondary antibodies. The blots were visualized using ECL reagents and imaged on a digital scanner.

### Quantitative RT-PCR analysis

Total RNA from HL60 or HL60/ADR cells was isolated using TRIzol reagent (Life Technologies, Carlsbad, CA, USA) and the RNA concentration was determined using a NanoDrop spectrophotometer (Thermo Fisher Scientific, Waltham, MA, USA). Quantitative RT-PCR was performed as we previously described [38]. Briefly, 0.5 μg of RNA was synthesized to cDNA by using a PrimeScript^TM^ RT Master Mix Kit (Takara, Tokyo, Japan) according to the manufacturer's instructions. The quantitative PCR reaction was carried out by using a CFX96^TM^ Real-Time PCR Detection System (BIO-RAD, Hercules, CA, USA). The sequences of p27 primers were 5′-TGCAACCGACGATTCTTCTACTCAA-3′ (sense) and 5′-CAAGCAGTGATGTATCTGATAAAC-3′ (antisense). GAPDH was used as a housekeeping gene to normalize RNA expression, with the primers as 5′-GCACCGTCAAGGCTGAGAAC-3′ (sense) and 5′-GGTGAAGACGCCAGTGGA-3′ (antisense). The relative gene expression levels were quantified by using the comparative Ct (ΔΔCt) method.

### Synergism assay

Isobologram analysis (Chou-Talalay model) was used to determine the type of interaction (synergistic, additive or antagonistic effect) occurring when ZSTK474 was administered in combination with the chemotherapeutic drugs. The combination index (CI) for the experimental combinations (with a ratio of 1:1 for ZSTK474 and the chemotherapeutic drugs) was calculated by using CalcuSyn software [39, 40]. CI > 1 indicates antagonism, CI = 1 indicates additive effect, and CI < 1 indicates synergism. All experiments were carried out in triplicate.

### Statistical analysis

Data were presented as mean ± standard deviation (SD), representative of at least three independent experiments. Student's *t-*test was carried out for statistical significance analysis with GraphPad Prism 5 (GraphPad, San Diego, CA, USA). A value of P < 0.05 was regarded as statistically significant.

## SUPPLEMENTARY FIGURES


