# Supplementary material for: Antiproliferative effect of ZSTK474 alone or in combination with chemotherapeutic drugs on HL60 and HL60/ADR cells
Source: Oncotarget. 2017 Mar 28;8(24):39064–76. doi: 10.18632/oncotarget.16589 (PMC5503595; doi:10.18632/oncotarget.16589)
Supplement: Supplementary file 1 [file oncotarget-08-39064-s001.pdf]

Antiproliferative effect of ZSTK474 alone or in combination with chemotherapeutic drugs on HL60 and HL60/ADR cells

SUPPLEMENTARY MATERIALS

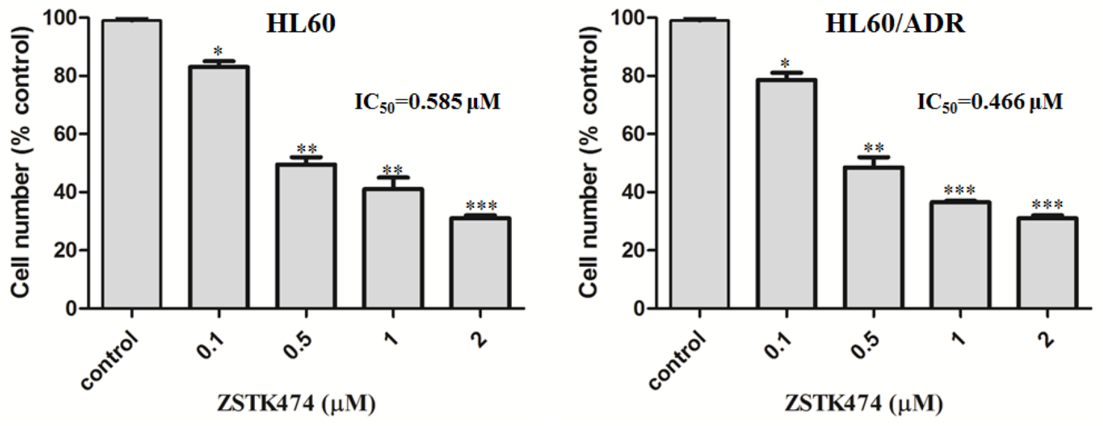

Supplementary Figure 1: Growth inhibitory effect of ZSTK474 on HL60 and HL60/ADR cells assessed by cell count assay. HL60 or HL60/ADR cells in 6-well plate were treated with ZSTK474 (0, 0.1, 0.5, 1, 2 µM). Forty eight hours later, cells were harvested, resuspended in 500 µl of serum free medium containing 0.1% trypan blue dye. The viable cells were counted under microscope. Cell number of each sample was expressed as the percentage relative to that of control. Data are presented as mean ± SD, representative of three independent experiments.*: $P<0.05$, **: $P<0.01$, ***: $P<0.001$, compared with control.
